# Animal heat activated cancer therapy by a traditional catalyst TiO_2_-Pd/graphene composites

**DOI:** 10.1038/s41598-020-72682-4

**Published:** 2020-09-25

**Authors:** Yanlong Yu, Pengchong Jiang, Yabin Yan, Hanbo Li, Lixin Zhang, Shan Jiang, Wensheng Yang, Yaan Cao

**Affiliations:** 1 Key Laboratory of Weak-Light Nonlinear Photonics, Ministry of Education, TEDA Applied Physics Institute and School of Physics, Nankai University, Tianjin, 300457 People’s Republic of China; 2 College of Chemistry, Jilin University, Changchun, 130012 People’s Republic of China; 3 Faculty of Mathematics and Physics, Huaiyin Institute of Technology, Huaian, 223003 China

**Keywords:** Nanoparticles, Catalytic mechanisms

## Abstract

Cancer therapy is one of the most important challenges in clinical medicine. So far different methods have been developed for cancer therapy, such as radiation therapy, surgery, chemotherapy and photodynamic therapy. Here we propose a new concept for cancer therapy, i.e., killing the cancer cells simply via reactive oxygen species (ROS) generated by TiO_2_-Pd/graphene composites. Activated by animal heat of 37 °C, the electrons in the valence band can be excited to the conduction band of TiO_2_ via the energy levels of Pd species and graphene, generating ROS without light irradiation or electric excitation. The tumors in BALB/c mice are successfully regressed at animal heat without any other external conditions, such as radiation, UV, visible and IR irradiation. Our results suggest that the design of animal heat activated cancer therapy is a feasible concept for practical applications of cancer treatments.

## Introduction

Cancer can be usually treated by surgery, chemotherapy, radiation therapy, hormonal therapy, targeted therapy (including immunotherapy such as monoclonal antibody therapy) and synthetic lethality^1–7^. The choice of therapy depends upon the location and grade of the tumor and the stage of the disease, as well as the general state of the patient (performance status). However, there are still serious side effects for these cancer treatment methods, which may cause damage on human bodies. Therefore, designing and developing new concept about the complete removal of the cancer without damage to the rest of the body is still the ideal goal of treatment.

During the last decades, many photosensitizers have been employed extensively for photodynamic therapy^8–10^. It is known that photodynamic catalyst can generate charge carriers and then produce reactive oxygen species (ROS) on surface under irradiation. These ROS can damage biological DNA and proteins, damaging and killing the cancer cells eventually^3,6^. In addition, photothermal treatment uses near-infrared absorbing nanoparticles to generate heat, resulting in thermal ablation to kill cancer cells. CuS@MSN based theranostic nanoparticles have been designed and synthesized for tumor vasculature targeting and photothermal therapy^2^. The Au–Cu nanocrystals exhibit a notable photothermal effect to kill cancer cells irradiated by a 808 nm laser^11^. However, irradiation necessarily used for photodynamic and photothermal cancer treatment is difficult to be applied in vivo system. Therefore, it is of great importance to design and develop new cancer therapy concepts without any additional irradiation.

Herein, we introduced a new concept, animal heat activated cancer therapy with a traditional catalyst. The ROS can be generated by TiO_2_-Pd/graphene without any external conditions, which could kill the cancer cells and regress the tumors in BALB/c mice under animal heat (37 °C), implying the feasibility of brand new concept for the cancer treatment. This concept may possess potential advantages for the practical cancer therapy, such as less body damage, selectivity on killing cancer cells and normal body cells.

## Results and discussion

**Figure 1:**
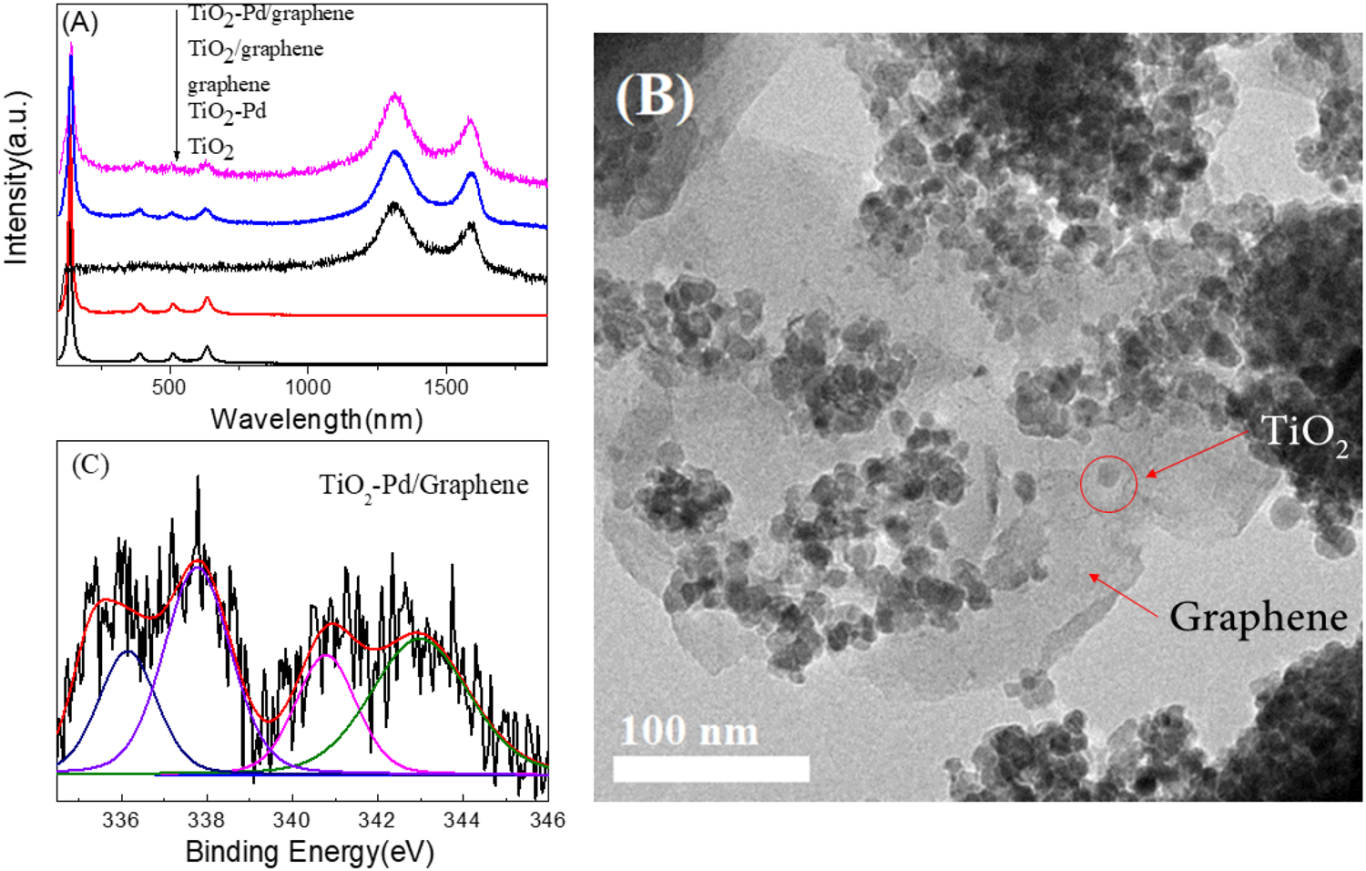
(**A**) Raman spectra of TiO_2_, graphene, TiO_2_/graphene, TiO_2_-Pd and TiO_2_-Pd/graphene samples. (**B**) HRTEM images of TiO_2_-Pd/graphene (**C**) XPS Pd 3d spectrum of TiO_2_-Pd/graphene.

Raman spectra, XPS and HRTEM are applied to investigate the structure of TiO_2_-Pd/graphene . As shown in Fig. 1A, the Raman peaks of anatase at 144 cm^−1^ (Eg), 194 cm^−1^ (Eg), 396 cm^−1^ (B1g), 516 cm^−1^ (A1g and B1g), and 638 cm^−1^ (Eg) are observed in TiO_2_ and TiO_2_-Pd samples. For TiO_2_/graphene and TiO_2_-Pd/Graphene, besides the Raman peaks of anatase, the peaks of reduced graphene at 1315 and 1585 cm^-1^ are detected^12,13^. Similar results are obtained from the XRD patterns (Fig. S1). No other XRD peaks, such as PdO can be observed. The cell volume and lattice parameters of TiO_2_-Pd x% and TiO_2_-Pd/Graphene x% derived from XRD data remain almost unchanged (Fig. S2 and Table S1) compared with TiO_2_. This suggests that Pd ions are not doped into TiO_2_ lattice in substitutional or interstitial mode, as the ionic radius of Pd^2+^ (86 pm) is much larger than that of Ti^4+^ ions (68 pm). Therefore, it can be deduced that the Pd ions might exist on the surface of TiO_2._ Moreover, according to the XPS results, the Cl 2*p*_3/2_ peaks (198.5 eV) for TiO_2_-Pd/Graphene (Fig. S3A) locates between that of TiCl_4_ (198.2 eV) and PdCl_2_ (198.9 eV), ascribed to the surface O–Pd-Cl structure^14^. Two pairs of doublet Pd 3d peaks can be observed for TiO_2_-Pd/Graphene (Fig. 1C). One Pd3d_5/2_ peak at 337.7 eV is attributed to –O–Pd–Cl structure (i.e., one Pd^2+^ ion is linked with one Cl^–^ ion and one unsaturated oxygen ion) on the surface. The other peak at 336.2 eV for Pd 3d_5/2_ is ascribed to –O–Pd–O– species on the surface (i.e., one Pd^2+^ ion is linked with two unsaturated oxygen ions, which has been confirmed by our previous work^14^). The molar ratio of Pd/Ti for TiO_2_-Pd and TiO_2_-Pd/graphene is 4.19%/100% and 4.14%/100%, respectively. Moreover, the C1*s* peak at 284.3 eV (Fig. S3B) is ascribed to the graphene. HRTEM image of TiO_2_-Pd/Graphene (Fig. 1B) confirms TiO_2_-Pd nanoparticles are attached on the surface of graphene. In addition, the peak of 1216 cm^-1^ in the FR-IR spectra of TiO_2_-Pd/Graphene is ascribed to vibration of C–O bond (Fig. S5). The XPS Ti 2*p* spectra (Fig. S24 and S25) also confirms the graphene and TiO_2_-Pd are connected via the Ti–O–C bonds.

**Figure 2:**
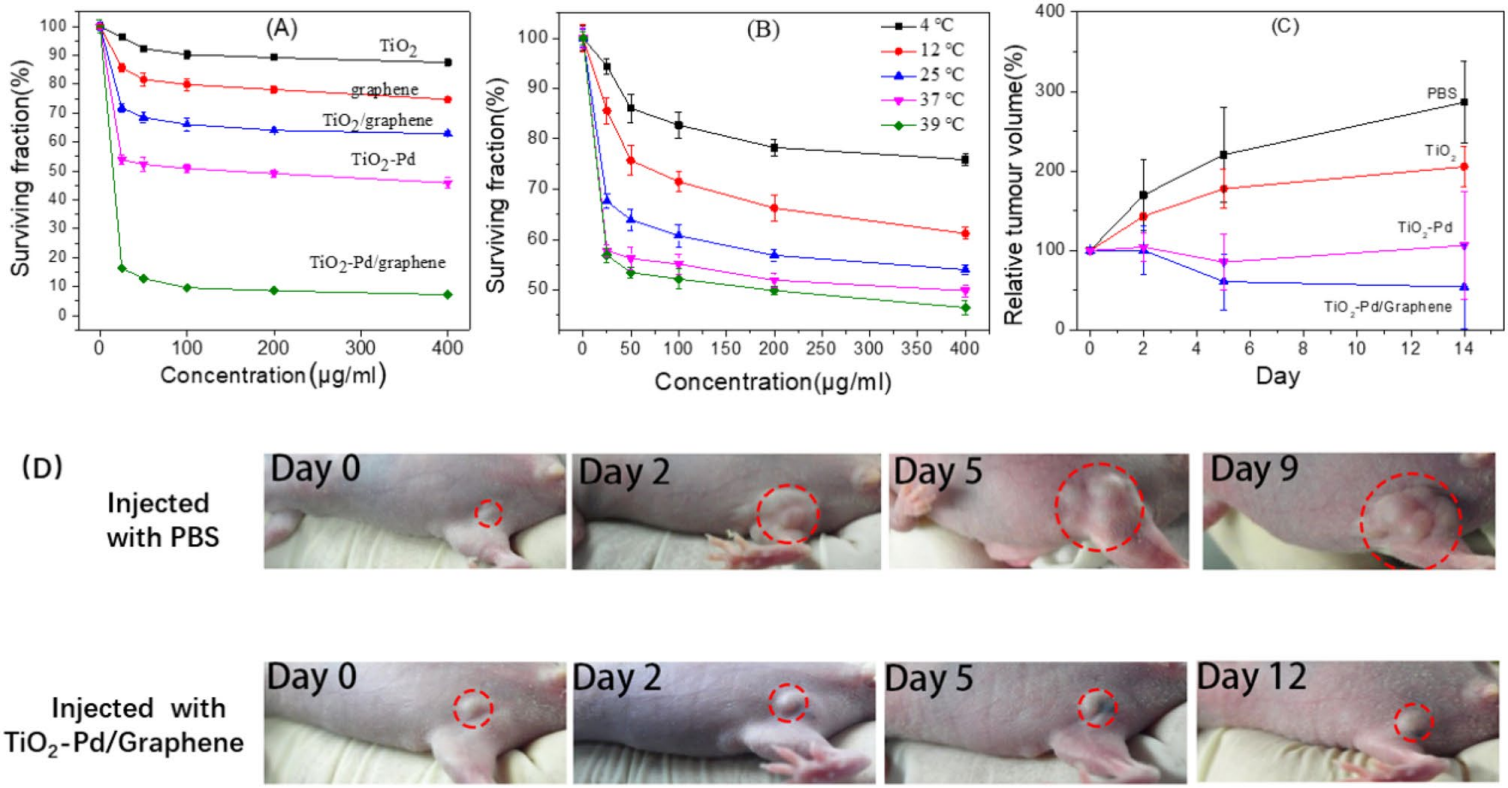
(**A**) Variations in surviving fraction of A549 cells with the concentraions of TiO_2_, graphene, TiO_2_/graphene, TiO_2_-Pd and TiO_2_-Pd/graphene at 37 °C for 16 h. (**B**) Variations in surviving fraction of A549 cells with the concentration of TiO_2_-Pd/graphene at different temperatures for 4 h. (**C**) Tumor volume growth curves on mice after treatment with different samples. Error bars were based on s.d. of 3 mice per group. (**D**) Representative photos of tumors on mice after various treatments indicated.

The surviving fraction of the A549 cells was measured by standard 3-(4,5-dimethylthiazol-2-yl)-2,5-diphenyltetrazolium bromide (MTT) assay to evaluate the ability of killing cancer cells for all samples at 37 °C, as shown in Fig. 2A. The surviving fraction of the A549 cells is about 96% for TiO_2_, 85% for graphene, 71% for TiO_2_/graphene, 53% for TiO_2_-Pd and 16% for TiO_2_-Pd/graphene, respectively, when the concentration is 25 μg/ml after 16 h of incubation at 37 °C. Moreover, it is found that the surviving fraction of cancer cells (16%) for TiO_2_-Pd/graphene is about 1/5 times as that for pure TiO_2_. With the increase of sample concentration, the performance of killing cancer cells falls in order of TiO_2_ < graphene < TiO_2_/graphene < TiO_2_-Pd < TiO_2_-Pd/graphene. Furthermore, we have also checked the surviving fraction of cancer cells treated with TiO_2_-Pd x% and TiO_2_-Pd/graphene x% and the surviving fraction is the lowest for TiO_2_-Pd1.5% and TiO_2_-Pd/graphene40% (Fig. S6). Furthermore, the increased Pd content (> 1.5%) and graphene content (> 40%) would inhibit the performance of killing cancer cells. Thus, the introduced Pd species and graphene into TiO_2_ system is the important key factor for the improved ability of killing cancer cells. The optical microscopy images of A549 cancer cells in the presence of samples (100 μg/mL) are shown in Fig. S9. Most of the A549 cells survive well in the blank experiment (without the addition of any samples) (Fig. S9a). The TiO_2_ can hardly influence the surviving of the cancer cells (Fig. S9b). It is found that the relative amount of healthy cancer cells decreased for TiO_2_/graphene (Fig. S9c) and TiO_2_-Pd (Fig. S9d). As we expected, seldom healthy cancers cell can be observed in Fig. S9e for TiO_2_-Pd/graphene samples, suggesting its high efficiency on killing cancer cells. These results are in good agreement with MTT results in Fig. 2A.

The effect of temperature on the surviving fraction of cancer cells treated with various concentration of TiO_2_-Pd/graphene for 4 h is evaluated in Fig. 2B. The surviving fraction of cancer cells for TiO_2_-Pd/graphene decreased with the increased system temperature. When the concentration of TiO_2_-Pd/Graphene is about 25 μg/mL, the surviving fraction of A549 cells are approximately 80%, 70%, 60%, 55%, and 50% at 4, 12, 25, 37 and 39 °C, respectively. Similar results are observed in the synovial cells (Fig. S7). This indicates the system temperature has great influence on the activity of killing cancer cells for TiO_2_-Pd/graphene, and TiO_2_-Pd/graphene can effectively inhibit the growth of cells at animal heat condition (37 °C).

Fig. S8 shows the relationship between the surviving fraction of the different (A549 and smooth muscle cells) cells and concentration of TiO_2_-Pd/graphene. It is found that only about 10% of cancer cells survived and 40% of smooth muscle cells survived at 37 °C for 16 h with a concentration of 25 μg/mL. This suggests a possible selectivity on cancer therapy for TiO_2_-Pd/graphene. It is expected that further investigation should be carried out by combining other techniques, such as targeted therapy, tumor injection, magnetic control to avoid the side effects on normal cells.

For in vivo animal heat activated therapy study, we employed subcutaneous 4T1 xenograft model in BALB/c mice to study the efficacy of animal heat activated cancer therapy (37 °C) after treatment by PBS (phosphate buffer saline), TiO_2_, TiO_2_-Pd and TiO_2_-Pd/graphene. Twelve tumor-bearing mice were randomly and evenly divided into four groups, which were intratumorally injected with PBS, TiO_2_ TiO_2_-Pd and TiO_2_-Pd/graphene, respectively. Figure 2C shows the thorough regression of tumor volume is observed only in the group with intratumoral injection of TiO_2_-Pd/graphene, while the volume of tumor increases for the group with PBS and TiO_2_ and remains unchanged for the group with TiO_2_-Pd. For the group with intratumoral injections of TiO_2_-Pd/graphene, the average relative tumor volume decreased to approximately 50% and for the group with intratumoral injections of TiO_2_-Pd, the average relative tumor volume remains almost unchanged. Figure 2D displays the representative photographs of tumors on mice that the intratumorally injected with PBS control and TiO_2_-Pd/graphene. The growth of tumor is inhibited when injected with TiO2-Pd/graphene. These experiment results indicate that TiO_2_-Pd/graphene is an effective functional material to inhibit cancer growth under animal heat (37 °C) without external irradiation or heating.

To get the physical insight of the band structure and density of states for TiO_2_-Pd/graphene, the theoretical calculation results of pure TiO_2_, TiO_2_-Pd and graphene are shown in Fig. S12–S15. Pure TiO_2_ indicates an indirect band gap of 2.46 eV (Fig. S12). The zero point corresponds to highest state level that electrons occupy. For TiO_2_-Pd, some new quasi-continuous energy levels in the band gap (Fig. S13 and S14) are attributed to the contribution of –O–Pd–Cl and O–Pd–O species. Meanwhile, it is obvious that the states of Pd 4*d* for –O–Pd–Cl and O–Pd–O species plays a great role to make the slightly connection between the conduction band and valence band. For TiO_2_-Pd (Fig. S13 and S14), the density of states for the conduction band and valence band overlapped with that for the energy levels of Pd species (Pd 4*d*), implying that the electrons in the valence band can transfer to the energy levels of Pd species (Pd 4*d*) or even conduction band excited by external energy. Moreover, the grapheme (Fig. S15) exhibit as a zero-gap semiconductor, implying electrons can transfer freely in the energy band of graphene^15^. These theory calculation results imply that the electrons can easily transfer between TiO_2_-Pd and graphene when TiO_2_-Pd/Graphene are excited by external energy such as animal heat. These thermal excited electrons and holes can transfer to the surface to further generate ROS. This theoretical calculation can be further demonstrated by the UV − Vis absorption spectra and XPS valence band spectra (Fig. S16–S18).

In Fig. S16, according to the above theory calculation, the stronger absorption in visible region for TiO_2_-Pd is caused by the electron transition from valence band to the quasi-continuous energy levels of Pd species (Pd 4*d*) or from the quasi-continuous energy levels of Pd species (Pd 4*d*) to the conduction band. Moreover, for TiO_2_/graphene and TiO_2_-Pd/graphene, the strong absorption in visible region is due to the continuous energy band structure of graphene.

**Figure 3:**
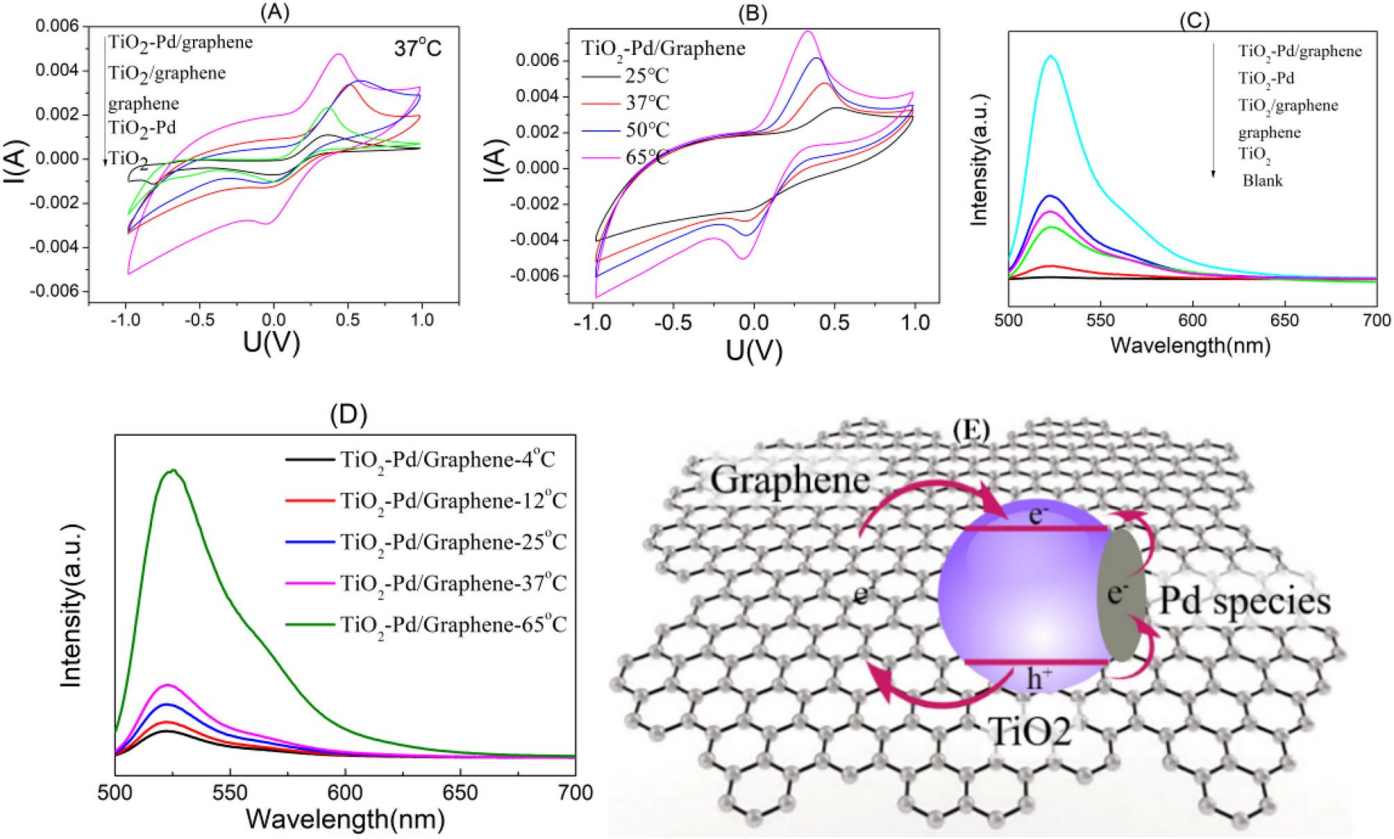
Cyclic voltammetry of all samples at 37 °C (**A**) and TiO_2_-Pd/graphene at different temperature (**B**). Verification of ROS generation ability for all samples at 37 °C (**C**) and TiO_2_-Pd/Graphene at different temperatures (**D**). Schematic band structure of TiO_2_-Pd/graphene composite (**E**).

For XPS valence band spectra (Fig. S18; the work function of instrument: 4.10 eV), the valence band top for pure TiO_2_ is 2.75 eV (2.35 eV; vs NHE), and the highest occupied state level for graphene is about 2.29 eV (1.89 eV; vs NHE). For TiO_2_-Pd, the valence band top is almost the same as that of TiO_2_, and a small hump from 2.75 to 0.10 eV (2.35 to − 0.3 eV vs NHE) is attributed to the energy level of Pd species (Pd 4*d*) occupied by electrons. For TiO_2_-Pd/graphene, the highest energy levels that electrons occupy extend to − 0.6 V (− 1.0 eV; vs NHE), owing to the synergetic effect of graphene and TiO_2_-Pd. These results imply the electrons in the valence band can transfer to the energy levels of Pd species (Pd 4*d*) or even the conduction band of TiO_2_ through the quasi-continuous energy levels freely and continuous energy band structure of graphene excited by the external energy. According to the results, the schematic band structure is determined for TiO_2_-Pd/graphene, as shown in Fig. 3E.

The Raman spectra, cyclic voltammetry curves and fluorescence spectra of the DCF are applied to investigate the relationship between the generation of ROS and temperature. The Raman spectra of TiO_2_-Pd and TiO_2_-Pd/graphene (Fig. S19) indicates the peak intensity was enhanced with the increase of temperature, suggesting an enhanced vibrating energy of lattice, benefiting the electrons’ transfer to surface of sample.

The cyclic voltammetry curves of pure TiO_2_, graphene, TiO_2_/graphene, TiO_2_-Pd and TiO_2_-Pd/graphene at 37 °C (in Fig. 3A) indicate the ability to give and receive electrons increases when Pd species and/or graphene are introduced into TiO_2_ system. For the cyclic voltammetry curves of the TiO_2_-Pd/graphene at different temperatures, the redox peaks of TiO_2_-Pd/graphene are improved with the increase of temperature (Fig. 3B). This suggests that TiO_2_-Pd/Graphene may exhibit enhanced ability to give and receive electrons under thermal excitation.

The amounts of ROS (^.^OH, O_2_^−^ , et al.) generated by TiO_2_, graphene, TiO_2_/graphene, TiO_2_-Pd and TiO_2_-Pd/graphene at 37 °C were evaluated by the photoluminescence (PL) intensity of the DCF (Fig. 3C). No PL peak is detected in the blank experiment. For TiO_2_ and graphene, a weak PL peak is found, suggesting a small amount of ROS formed. Moreover, for TiO_2_/graphene and TiO_2_-Pd, the PL intensity further increases, suggesting more ROS can be generated than TiO_2_. The PL intensity for TiO_2_-Pd/graphene is the strongest among all the samples, indicating TiO_2_-Pd/graphene is most effective to generate ROS among all the samples at 37 °C. Figure 3D shows generation amount of ROS for TiO_2_-Pd/graphene at different temperatures. It is noted that the PL intensity significantly increases with the increase of temperature, indicating the increased temperature is benefit for the generation of reactive oxidation species. This also confirms that the electrons and holes in TiO_2_ can be excited by heat and transfer to surface to form ROS.

Based on the aforementioned discussion, the mechanism of generating ROS (such as ·OH, O_2_^−^) and kill the cancer cells for all samples can be explained using the schematic band structure shown in Fig. 3E. The electrons in the conduction band (− 0.45 eV, vs NHE) excited by animal heat (37 °C), whose potential is more negative than that of O_2_/O_2_^−^ (− 0.33 eV, vs NHE), can transfer to the surface and directly captured by the adsorbed O_2_ molecules on the surface to form O_2_^−^ active species as shown in Eq. (1)^16–18^. The holes in the valence band (+ 2.65 eV, vs NHE), whose potential is more positive than H_2_O /H^+^ (0.82 V, vs NHE), are captured by surface absorbed H_2_O molecules to form O_2_ and H^+^, which can further react with thermal electrons to produce the hydroxyl free radical OH·as shown in Eqs. (2), (3) and (4)^16–18^.

1 $$ {\text{O}}_{{\text{2}}}  + {\text{ e }} = {\text{ O}}_{{\text{2}}}^{-}  $$

$$ {\text{E}}^{{\text{o}}} _{{{\text{redox}}}}  =  - 0.{\text{33 V vs NHE}} $$

$$ {\text{2H}}_{{\text{2}}} {\text{O }} + {\text{ 4h}}^{ + }  \to {\text{ O}}_{{\text{2}}}  + {\text{ 4H}}^{ + } $$

$$ {\text{E}}^{{\text{o}}} _{{{\text{redox}}}}  = {\text{ }}0.{\text{82 V vs NHE}}$$

2 $$ {\text{O}}_{{\text{2}}}  + {\text{ 2H}}^{ + }  + {\text{ 2e }} = {\text{H}}_{{\text{2}}} {\text{O}}_{{\text{2}}}  $$

3 $$ {\text{H}}_{{\text{2}}} {\text{O}}_{{\text{2}}}  + {\text{ e }} = {\text{ OH}}^{ - }  + {\text{ HO}}^{\cdot}  $$

4 $$ {\text{OH}}^{ - }  + {\text{ h}}^{ + }  = {\text{ HO}}^{\cdot}  $$

For pure TiO_2_, electrons can hardly be excited to the conduction band by the animal heat (37 °C), leading to a poor killing cancer cell activity, because of large band gap (3.0 eV). For TiO_2_-Pd, a small amount of electrons can be excited by the animal heat and transfer to the conduction band through the quasi-continuous energy levels of Pd species, leaving holes in the valence band of TiO_2_. For TiO_2_/graphene, the electrons could also be excited by the animal heat and transfer to the conduction band of TiO_2_ through the continuous energy levels of graphene, leaving holes in the valence band of TiO_2_. The electrons and holes would react with adsorbed O_2_ and H_2_O molecules on surface to form a small amount of ROS, which would kill the cancer cells directly. For TiO_2_-Pd/graphene, the electrons in the valence band can accept the energy from the animal heat and transfer to the conduction band through the quasi-continuous energy levels of Pd species and the continuous energy levels of graphene, generating more charge carriers than TiO_2_-Pd and TiO_2_/graphene. Therefore, more ROS can be produced and the TiO_2_-Pd/graphene exhibited the better capability of killing cancer cells than the other samples.

In summary, we have demonstrated a new concept for developing high efficient animal heat activated cancer treatment for TiO_2_-Pd/graphene. The electrons and holes can be excited through the energy levels of Pd species and graphene at animal heat, generating ROS which can further kill the cancer cells. This may afford a feasible and efficient approach for cancer therapy application, without any other external conditions such as radiation, UV, visible and IR irradiation that may cause serious body damage.

## Methods

### Catalyst preparation

#### *Synthesis of Pd-TiO*_*2*_

All chemicals used were of analytical grade and the water was deionized water (> 18.2 MΩ cm). At room temperature, certain volume of PdCl_2_ (0.1036 mol/L) solution were mixed with 40 mL of ethanol. Then 1 mL of HCl solution (12 mol/L) and 12 mL of Ti(OC_4_H_9_)_4_ was added dropwise into the mixture under vigorous stirring. The mixture was stirred until the formation of TiO_2_ gel, followed by being aged for 24 h. The obtained gels were dried at 373 K for 12 h and annealed at 723 K in a muffle for 2.5 h. The resultant samples were denoted as Pd-TiO_2_. Pure TiO_2_ was prepared using the same procedure, while without the addition of PdCl_2_ solution. Unless stated otherwise, the nominal molar ratio of Pd^2+^ to Ti^4+^ is fixed at 1.5% in the precursor and. For comparison, other molar ratios were also used for Pd^2+^ to Ti^4+^ (such as 0.5%, 1.0%, 2.0%, 2.5% and 3.0%).

#### Synthesis of graphene oxide (GO)

GO was prepared from crystalline flake graphite powder according to the modified method reported by Hummers and Offeman^19^. In brief, 10 g of graphite powder and 5 g of NaNO_3_ were added into 230 mL of cooled (273 K) concentrated H_2_SO_4_. Then, 30 g of KMnO_4_ was added gradually with continuous stirring and cooling, and the temperature of the mixture was maintained below 293 K. After the ice bath was removed, the mixture was stirred at 308 K for 30 min. 460 mL of distilled water was added slowly to cause an increase in temperature to 371 K, and the mixture was maintained at that temperature for 15 min. The reaction was terminated by addition of 1.4 L of distilled water followed by 25 mL of 30% H_2_O_2_ aqueous solution. The solid product was separated by centrifugation and washed repeatedly with 5% HCl solution (2L) and deionized water until sulfate anion could not be detected with BaCl_2_. The resultant solid was dried in vacuum at 323 K to obtain GO.

#### Preparation of TiO_2_-Pd/grapheme

GO was first dissolved in deionized water by ultrasonic treatment for 20 min. Then, Pd-TiO_2_ was added into the GO colloidal solution and the mixture was ultrasonic for another 1 h to obtain a homogeneous suspension. The resultant composite was collected by drying at 333 K and then triturated to powder in an agate mortar. Finally, the powder was calcined at 300 °C for 2 h under Ar atmosphere. The resulting products wereTiO_2_-Pd/graphene.

#### Characterizations

The XRD patterns were acquired using a Rigaku D/max 2500 X-ray diffraction spectrometer (Cu Kα, λ = 1.54056 Å). The average crystallite size was calculated according to the Scherrer formula (*D* = k λ/*B* cosθ). Raman spectra were taken on a Renishaw inVia Raman microscope by using the 785 nm line of a Renishaw HPNIR 785 semiconductor laser. The Fourier Transform Infra-Red (FT-IR) spectra were recorded for KBr disks containing the powder sample with an FT-IR spectrometer (MAGNA-560). The BET surface areas of the samples were determined by nitrogen adsorption–desorption isotherm measurements at 77 K (Micromeritics Automatic Surface Area Analyzer Gemini 2360, Shimadzu). XPS measurements were carried out by using a Thermo ESCALAB 250 spectrometer with an Al Kα monochromator source and all the binding energies were calibrated to the adventitious C1s peak at 284.8 eV. Diffuse reflectance UV–visible (UV–Vis) absorption spectra were recorded on a UV–Vis spectrometer (U-4100, Hitachi). ROS in the presence of samples was qualitatively detected by the H2DCF-DA assay. Photoluminescence (PL) spectra were acquired by using the 325 nm line of a nano-second Nd: YAG laser (NL303G) as excitation source. The experimental setup consists of a spectrometer (Spex 1702), a photomultiplier tube (PMT, Hamamatsu R943), a lock-in amplifier, and a computer for data processing. The cyclic coltammetry curves were measured using an electrochemical workstation (CorrTest, Wuhan, Inc.) in a conventional three-electrode cell at different temperature. The samples/ITO is used as working electrode, Pt is used as counter electrode and the saturated calomel electrode (SCE) was used as reference electrode. 0.5 mmol/L K_4_[Fe(CN)_6_]^+^ + 0.05 mmol/L K_3_[Fe(CN)_6_]^+^ + 0.1 mol/L KCl aqueous solution was used as electrolyte.

The generation ability of reactive oxygen species (ROS) for thermal catalysts can be estimated by measuring the fluorescent intensity of 2′,7′-dichlorofluorescein (DCF). The 2′,7′-dichlorodihydrofluorescein (DCFH, non-fluorescent) can rapidly react with ROS in the thermal catalysis system to form 2′,7′-dichlorofluorescein (DCF, fluorescent). By measuring the fluorescent intensity of DCF, the generation ability of ROS can be determined for thermal-catalyst. Experimental process is as follow: 5 mg of catalysts were added into 5 ml centrifuge tube, then 1 ml working solution (975 μl of diluents and 25 μl staining fluid containing DCFH) was added. The mixture was shock and heated at different temperature for 30 min (room temperature, 65 °C). Then, the mixture was centrifuged and the supernatant was taken to detect the fluorescent intensity. The exciting wavelength for exaction is 491nm^20^.

### Apoptosis assay

Human Lung Carcinoma cells (A549), smooth muscle cells and synovial cells were cultured in RPMI 1640 medium in 96-well plates, containing 10% fatal calf serum (FCS) in a humidified incubator with an atmosphere of 5% CO_2_ in air at 37 °C. The cell density was 2 × 10^4^ cells per well. After being seeded for 24 h, the media were replaced by culture media containing a series of TiO_2_, Pd-TiO_2_ and TiO2-Pd/Graphene nanoparticles with increasing concentrations in RPMI 1640 medium and then the plates were placed into the humidified incubator. After another 16 h for the interaction between the cancer-cells and the nanocomposite particles, cell viabilities were measured by the standard MTT assay, a colorimetric assay based on the ability of viable cells to reduce 3-[4,5-dimethylthiazol-2-yl]-2,5-diphenyltetrazolium bromide. The survival rate and the error bar are shown in Fig. 2B (the case of incubating with nanocrystals only).

#### In vivo thermal therapy

BALB/c mice bearing A549 lung carcinoma tumors were intratumorally injected with TiO_2_, Pd-TiO_2_ or graphene /Pd-TiO_2_ (80 ul of 4 mg/ml solution for each mouse), respectively. The images were taken by an high definition camera. The tumor sizes were measured by a caliper every other day and calculated as volume = (tumor length) × (tumor width)^2^/2. Relative tumor volumes were calculated as V/V0 (V0 is the tumor volume when the treatment was initiated). The animal protocol in this study conformed to the Guide for the Care and Use of Laboratory Animals (the Guide, NRC 2011), and it was also approved by the Institutional Animal Care and Use Committee at Nankai University (Approval ID 201009080081).

### Supporting information

Thermal catalytic activity, XRD, XPS Cl 2*p*, PDOS, Absorption spectra, XPS valence band spectra, Raman spectra, PL spectra and thermal excited PL spectra.

## Supplementary information

Supplementary information.
