# Supplementary material for: Animal heat activated cancer therapy by a traditional catalyst TiO2-Pd/graphene composites
Source: Sci Rep. 2020 Sep 25;10:15823. doi: 10.1038/s41598-020-72682-4 (PMC7519649; doi:10.1038/s41598-020-72682-4)
Supplement: Supplementary file 1 — Supplementary information. [file 41598_2020_72682_MOESM1_ESM.docx]

Supplementary Information for

Animal heat activated cancer therapy by a traditional catalyst TiO_2_-Pd/graphene composites

Yanlong Yu^1^, Pengchong Jiang^1^, Yabin Yan^1^, Hanbo Li^1^, Lixin Zhang^1^, Shan Jiang^2^ Wensheng Yang^2^ & Yaan Cao^1,^*

^1^Key laboratory of Weak-Light Nonlinear Photonics, Ministry of Education, TEDA Applied Physics Institute and School of Physics, Nankai University, Tianjin 300457, China

^2^College of Chemistry, Jilin University, Changchun 130012, P.R. China

Fig. S1. XRD patterns of TiO_2_, TiO_2_-Pd, TiO_2_/graphene and TiO_2_-Pd/graphene. Inset is the enlarged XRD peaks of crystal plane (101).

Table S1. Cell parameters, cell volume and crystallite size of TiO_2_, Pd-TiO_2_ and GO/Pd-TiO_2_ samples, which were derived from XRD data given in Fig. S1.

| Sample | a=b (Å) | c (Å) | Cell volume (Å^3^) | Crystallite size(nm) | BET specific surface area(m^2^/g) |
| --- | --- | --- | --- | --- | --- |
| TiO_2_ | 3.789 | 9.509 | 136.5 | 12.6 | 63.1 |
| TiO_2_-Pd | 3.791 | 9.510 | 136.8 | 10.7 | 82.5 |
| TiO_2_/graphene | 3.790 | 9.507 | 136.6 | 10.5 | 84.6 |
| TiO_2_-Pd/graphene | 3.792 | 9.512 | 136.6 | 9.3 | 93.7 |

The diffraction peaks corresponding to crystal planes (101) and (200) in the XRD patterns were selected to determine the cell parameters and cell volumes of the samples using Scherrer’s formula.

Fig. S2. XRD patterns of TiO_2_, X%Pd-TiO_2_ and Y%graphene/Pd-TiO_2_.

Fig. S3. XPS spectra for TiO_2_ and TiO_2_-Pd/Graphene. (A) Cl2p, (B) C 1s.


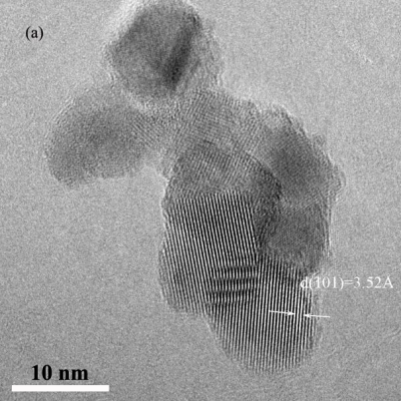

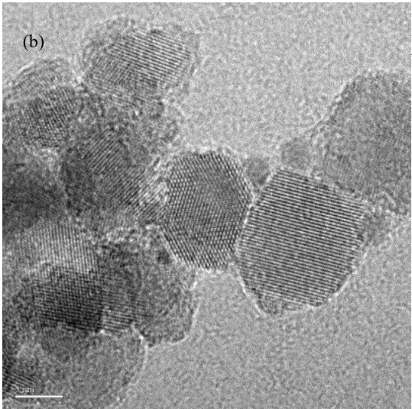

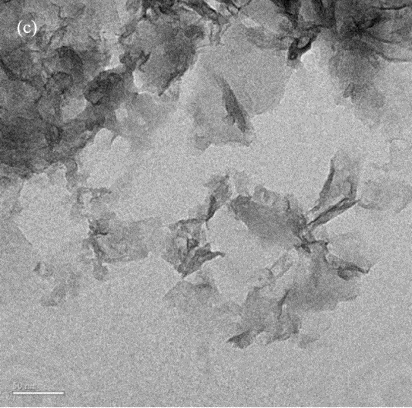


Fig. S4. TEM images of TiO_2_ (a), TiO_2_-Pd (b) and Graphene (c).

Fig. S5. FR-IR spectra of TiO_2_-Pd/Graphene

Fig. S6. Surviving fraction of A549 cancer cells as a function of concentration in the presence of all samples.

Fig. S7. Surviving fraction of synovial cells as a function of concentration for (A) TiO2-Pdx% for 4 h at 37^o^C; (B) TiO2-Pd/graphene x% for 4 h at 37^o^C; (C) TiO2-Pd/graphene at different temperatures for 16 h; (D) TiO2-Pd/graphene for different time.

Fig. S8. Surviving fraction of A549 and smooth muscle cells as a function of concentration of TiO_2_-Pd and TiO_2_-Pd/graphene.


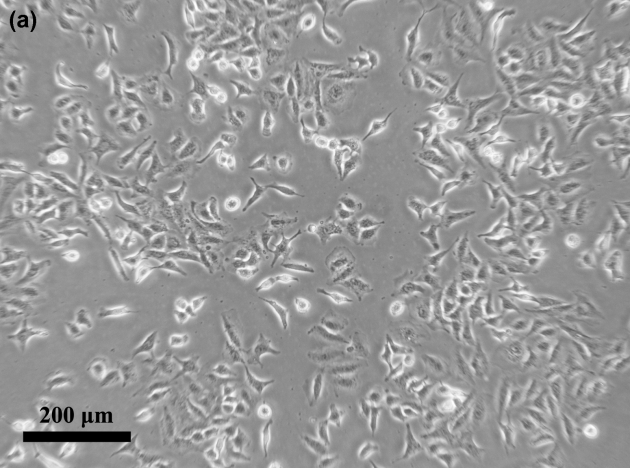

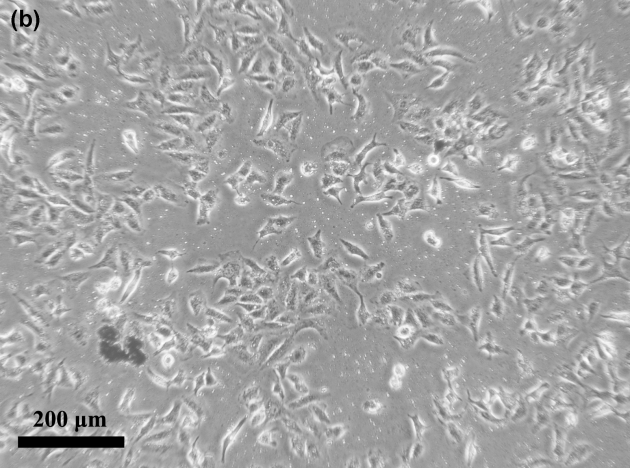


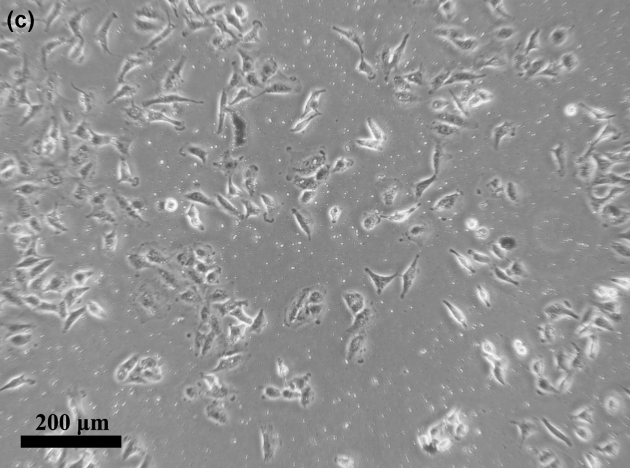

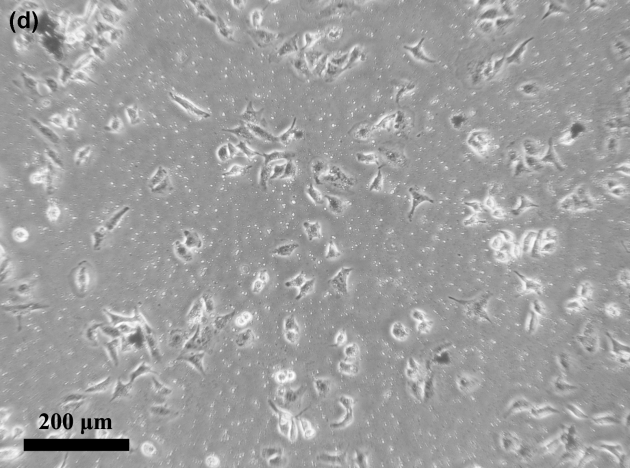


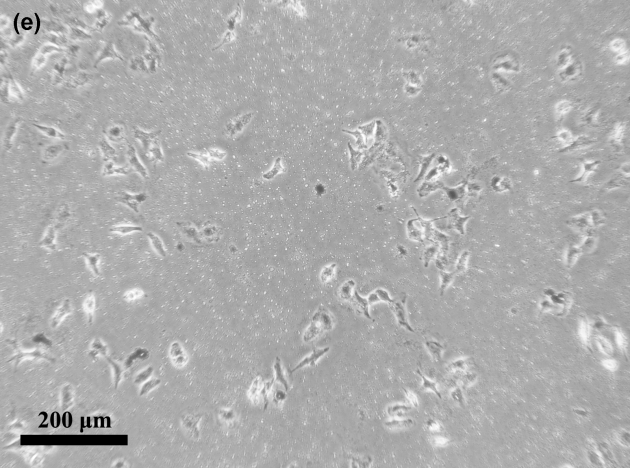


Fig. S9 optical microscopy pictures of A549 cancer cells treated in the presence of (a)TiO_2_, (b)Graphene, (c)TiO_2_/graphene, (d)TiO_2_-Pd and (e)TiO_2_-Pd/graphene (100 μg/mL) for 16 hours at 37^o^C.


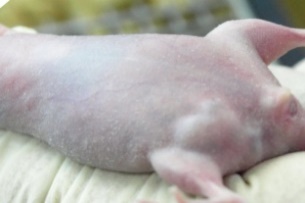

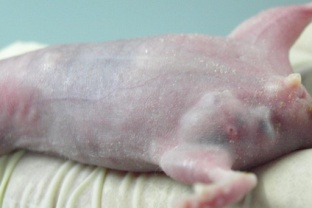

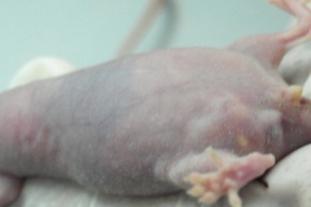

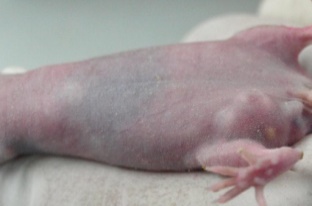

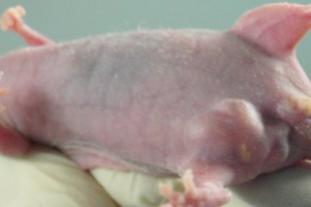


Fig. S10 Representative photos of tumors on mice injected with TiO_2_ after 0,2,4,5,9 days


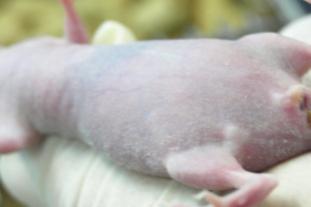

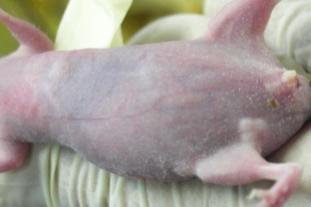

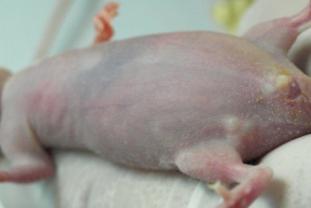

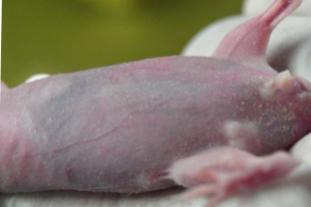

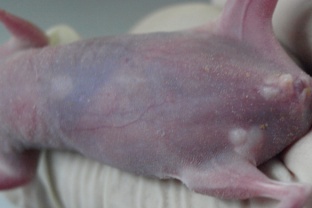


Fig. S11 Representative photos of tumors on mice injected with TiO2-Pd after 0,2,4,5,12 days

The calculation of the surface TiO2 structure were based on the density functional theory, as implemented in the VASP code. The ex-change-correlation energy was represented by the generalized-gradient approximation (GGA) of Perdew, Burke and Ernzerhof (PBE). An anatase TiO2 model of 48 atoms with an exposed (101) facet is created. The vacuum lamp is set at 20 Ai.

The valence electronic configurations for the O, Ti, Pd and Cl atoms were 2s^2^2p^4^, 3s^2^ 3p^6^3d^2^4s^2^, 4d^10^ and 3s^2^3p^5^, respectively. The plane wave energy cutoffs were taken to be 520 eV and the k-point set is 2 ×2 × 2. Compared with the experimental results, the theoretical calculation usually results in an underestimated band gap, caused by the shortcoming of the exchange–correction functional in describing the excited states


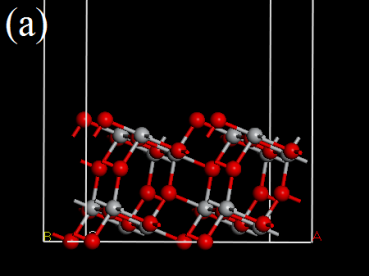


Fig. S12. Theoretical calculated band structure and density of states of pure TiO_2_.


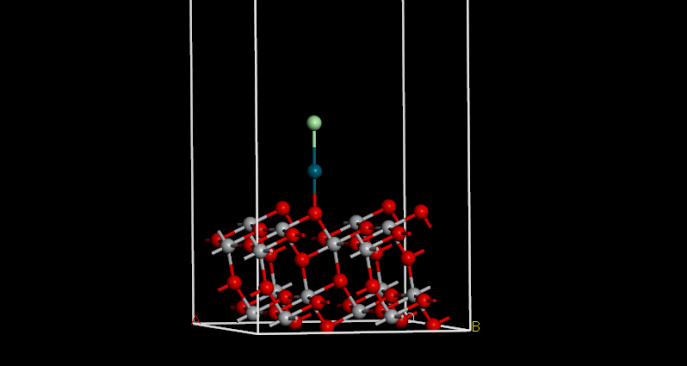


Fig. S13. Theoretical calculated band structure of TiO_2_-Pd (O-Pd-Cl)


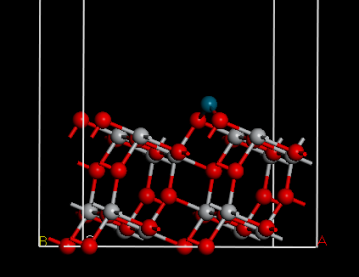


Fig. S14. Theoretical calculated band structure of TiO_2_-Pd (O-Pd-O)


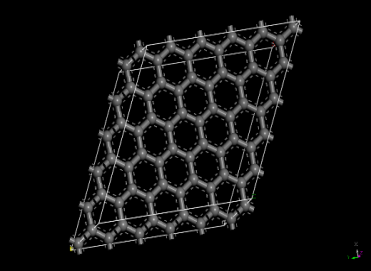


Fig. S15 Theoretical calculated band structure of graphene

Fig. S16. Absorption spectra of TiO_2_, TiO_2_-Pd, Graphene, TiO_2_/Graphene and TiO_2_-Pd/Graphene

Fig. S17. Absorption spectra of TiO_2_-PdX% and TiO_2_-Pd/Graphene Y%.

Fig.S18.XPS valence band spectra of TiO_2_, TiO_2_-Pd, Graphene, and TiO_2_-Pd/Graphene.

Fig. S19. Raman spectra of TiO_2_-Pd and TiO_2_-Pd/graphene at different temperatures.

Fig. S20. Cyclic voltammetry of TiO_2_ and TiO_2_-Pd.

Fig. S21. Verification of ROS generation ability for all samples.

Fig. S22. Verification of ROS generation ability for TiO2-Pd at different temperatures.


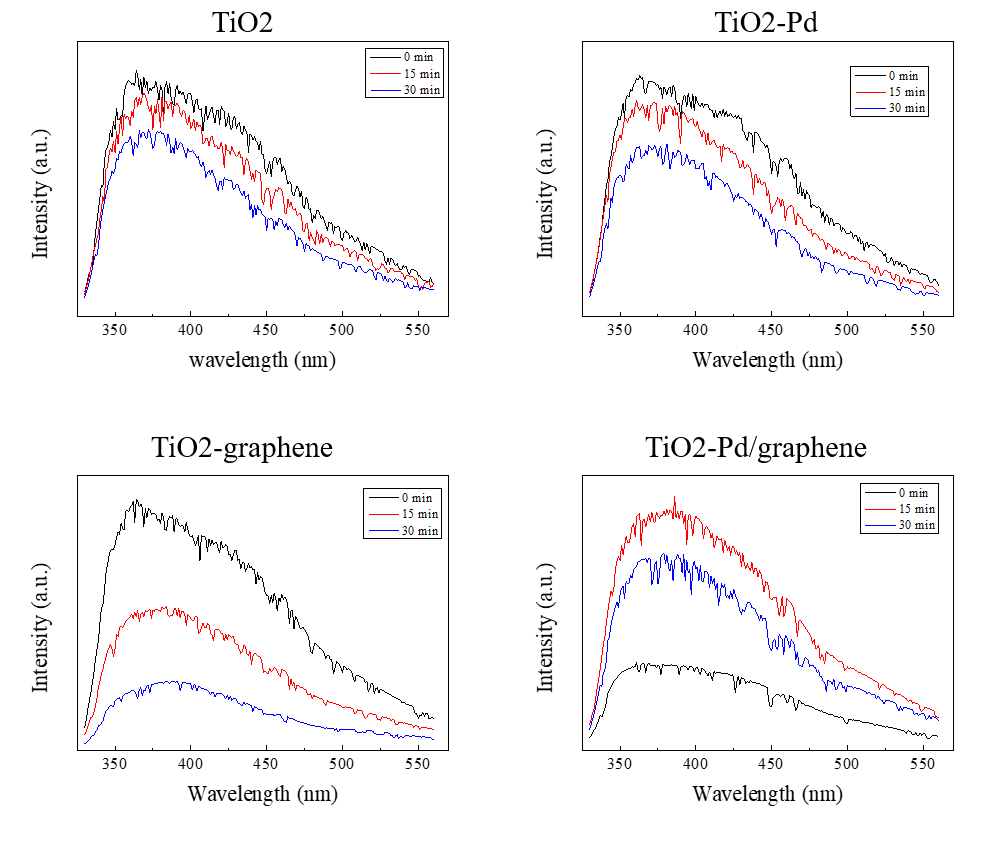


Fig.S23. PL spectral changes during irradiation in 0.5mmol/L terephthalic acid and 2mmol/L NaOH solution with all sample at 65^o^C for 0 min, 15 min and 30 min.

The generation of hydroxyl radicals was measured as follow: 5 mg of samples were added in 40 ml of mixed solution of 5% serum, 0.5mmol/L terephthalic acid and 2 mmol/L NaOH. After thermal treatment for 0 min, 15min and 30 min, 3 mL of supernatant was taken for measuring the PL intensity. The PL spectra were measured by the same instrument using the 315 nm line as the excitation source. It is noted that only TiO2-Pd/graphene samples can generate hydroxyl radicals at 65 ^o^C. This suggest the strong ability of generating hydroxyls radicals for TiO2-Pd/graphene.
